# Identifying Like-Minded Audiences for Global Warming Public Engagement Campaigns: An Audience Segmentation Analysis and Tool Development

**DOI:** 10.1371/journal.pone.0017571

**Published:** 2011-03-10

**Authors:** Edward W. Maibach, Anthony Leiserowitz, Connie Roser-Renouf, C. K. Mertz

**Affiliations:** 1 Center for Climate Change Communication, George Mason University, Fairfax, Virginia, United States of America; 2 Yale Project on Climate Change, Yale University, New Haven, Connecticut, United States of America; 3 Decision Research, Eugene, Oregon, United States of America; Umea University, Sweden

## Abstract

**Background:**

Achieving national reductions in greenhouse gas emissions will require public support for climate and energy policies and changes in population behaviors. Audience segmentation – a process of identifying coherent groups within a population – can be used to improve the effectiveness of public engagement campaigns.

**Methodology/Principal Findings:**

In Fall 2008, we conducted a nationally representative survey of American adults (*n* = 2,164) to identify audience segments for global warming public engagement campaigns. By subjecting multiple measures of global warming beliefs, behaviors, policy preferences, and issue engagement to latent class analysis, we identified six distinct segments ranging in size from 7 to 33% of the population. These six segments formed a continuum, from a segment of people who were highly worried, involved and supportive of policy responses (18%), to a segment of people who were completely unconcerned and strongly opposed to policy responses (7%). Three of the segments (totaling 70%) were to varying degrees concerned about global warming and supportive of policy responses, two (totaling 18%) were unsupportive, and one was largely disengaged (12%), having paid little attention to the issue. Certain behaviors and policy preferences varied greatly across these audiences, while others did not. Using discriminant analysis, we subsequently developed 36-item and 15-item instruments that can be used to categorize respondents with 91% and 84% accuracy, respectively.

**Conclusions/Significance:**

In late 2008, Americans supported a broad range of policies and personal actions to reduce global warming, although there was wide variation among the six identified audiences. To enhance the impact of campaigns, government agencies, non-profit organizations, and businesses seeking to engage the public can selectively target one or more of these audiences rather than address an undifferentiated general population. Our screening instruments are available to assist in that process.

## Introduction

Global warming is a classic “wicked problem.” [1] Wicked problems have no easy solutions in that they are beyond the capacity of any one organization to solve, and there is disagreement among organizations about both the causes and the best means by which to solve the problem [2]. Managing wicked problems requires working successfully within and across organizational boundaries, engaging citizens and other stakeholders in policy-making and implementation of those policies, and ultimately changing the behavior of groups of citizens or all citizens [2], [3].

Successfully mitigating and adapting to global warming will require significant modifications in public policy and population behavior [4]. Public engagement campaigns are an important strategy to encourage population behavior change and build support for appropriate public policies [5]–[8]. Many factors limit the success of engagement campaigns, however, some of them inherent (e.g., the myriad influences on human behavior that are largely beyond the reach of a communication campaign) [5], [7], [8] and others situational (e.g., the tendency of governments to prematurely terminate public engagement campaigns) [7], [9].

Although the research literature on global warming communication campaigns is relatively new and not yet well developed [5], [7], other fields including commercial marketing [10], social marketing [11], public health [12] and political science [13] offer considerable research on the attributes of effective public engagement campaigns. Audience segmentation is one of the methods widely supported in all of these diverse research literatures.

Audience segmentation is a process of identifying groups of people within a larger population who are homogeneous with regard to critical attributes (e.g., beliefs, behaviors, political ideology) that are most relevant to the objectives of a public engagement campaign (e.g., product sales, consumer boycotts, political participation) [14]. Audience segmentation research – conducted insightfully – provides organizations with an important strategic planning asset: empirical information about how best to focus the organization's limited resources, both human and financial, to advance its objectives [15]. For example, a smaller audience segment whose members are willing to behave in ways sought by the organization may be a more productive target than a larger, less predisposed audience segment.

The principal aim of our current research was to identify audience segments within the American adult population that could be considered as potential targets for global warming public engagement campaigns. The nature of the global warming public engagement challenge – i.e., the need to build public understanding and support for appropriate public policies, and to change the behavior of large numbers of people – necessitated that we adapt and extend previously used segmentation methods.

Specifically, there is strong precedent in the research literature for segmenting audiences based on what people are doing (i.e., behaviors) and why (i.e., motivations) [16]–[19]. That method is well suited to population behavior change campaigns (e.g., smoking cessation campaigns), but it largely ignores a second potential focus for global warming public engagement campaigns: building public understanding of and support for appropriate public policies. Here, we extend the method of segmenting audiences based on what people are doing and why to also include people's policy preferences as an additional dimension in the analysis.

The other aim of our research was to develop an easily implemented, survey-based identification tool that can be used to identify the audience segments in independent population samples with acceptable levels of accuracy. Such a tool will enable social science researchers and public engagement campaign planners to further study the audience segments identified in our research, and to test public engagement methods with them. We believe that both aims of our research were achieved.

## Results

We conducted a nationally representative survey of adults (n = 2,164) and used three major categories of variables as inputs into a segmentation analysis: global warming motivations, behaviors, and policy preferences. The global warming motivations category included two distinct sub-categories: beliefs about global warming and degree of involvement in the issue. We measured a total of 36 variables across these four categories (Tables 1, 2, 3 and 4). To maximize the practical value of the segmentation findings, we limited the analysis to five, six and seven segment solutions. As described in the Methods section below, we determined that the six-segment solution was optimal.

**Table 1:** Global Warming Beliefs by Audience Segment.

Survey Questions	Audience Segment						Scale Points
	Alarmed	Con-cerned	Cautious	Dis-engaged	Doubtful	Dis-missive	
1. & 1a. Certainty global warming is occurring	8.70	7.92	6.54	5.91	5.06	3.06	9
2. Human causation (% agree)	88	79	49	39	8	1	---
3. Scientific consensus (% agree)	80	64	37	23	11	8	---
4. Personal risk	3.09	2.59	1.90	2.75	1.29	1.02	4
5. Risk to future generations	3.98	3.78	2.96	4.00	1.89	1.04	4
6. Risk to plant & animal species	3.97	3.78	3.00	3.40	1.94	1.12	4
7. Timing of harm to Americans	5.46	4.83	3.53	3.85	1.77	1.01	6
8. Ability of humans to successfully mitigate warming	3.90	3.74	3.45	3.38	2.33	1.57	5
9. Actions of individual can make a difference	3.36	3.07	2.69	2.76	2.35	1.86	4
10. Technological optimism	1.70	2.05	2.32	2.03	2.38	2.33	4
11. Perceived impact of own mitigation actions	2.94	2.72	2.31	2.41	1.53	1.02	4
12. Impact of own actions if widely adopted in United States	3.69	3.48	3.01	2.90	1.94	1.10	4
13. Impact of own actions if widely adopted in modern industrialized countries	3.84	3.76	3.34	3.24	2.27	1.18	4

(*p*<.001 for all differences).

**Table 2:** Global Warming Issue Involvement by Audience Segment.

Survey Questions	Audience Segment						Scale Points
	Alarmed	Con-cerned	Cautious	Dis-engaged	Doubtful	Dis-missive	
14. Rating of global warming (good = 1 to bad = 6)	5.72	5.31	4.35	4.04	3.66	3.19	6
15. Worry about global warming	3.65	3.08	2.44	2.31	1.56	1.12	4
16. Thought given to global warming	3.65	2.75	2.22	1.71	2.19	2.82	4
17. Need for information (4 = low need)	2.74	2.16	1.89	1.60	2.50	3.58	4
18. Personal importance of issue	4.44	3.39	2.59	2.54	1.81	1.38	4
19. Unwilling to change opinion	3.77	2.95	2.41	2.16	3.02	3.69	5
20. Personally experienced global warming	2.92	2.26	1.95	1.96	1.52	1.19	4
21. Global warming discussion frequency	3.02	2.36	1.86	1.29	1.88	2.05	4
22. Friends share views on global warming	3.59	2.71	2.21	1.65	2.85	3.61	5

(*p*<.001 for all differences).

**Table 3:** Global Warming and Energy Use Behaviors by Audience Segment.

Survey Questions	Audience Segment							Scale Points
	Alarmed	Con-cerned	Cautious	Dis-engaged		Doubtful	Dis-missive	
14. Contacted govt. officials re mitigation	1.53	1.11	1.07		1.07	1.06	1.00	5
15. Rewarded companies that reduced emissions	3.34	2.18	1.50		1.38	1.31	1.19	5
16. Intend to reward companies that reduce emissions	2.76	2.51	2.17		2.14	2.06	1.92	3
17. Punished companies that are not reducing emissions	3.14	1.92	1.32		1.28	1.18	1.08	5
18. Intend to punish companies that are not reducing emissions	2.73	2.51	2.13		2.18	2.03	1.79	3
19. Stage of change for lowering thermostat in winter	7.02	6.50	5.99		5.74	6.21	6.18	10
20. Stage of change for using public transportation or car pool	3.92	3.06	2.74		3.14	2.11	2.27	10
21. Stage of change for walking/biking instead of driving	4.73	3.49	3.14		2.59	2.68	2.72	10
22. Stage of change for CFL use	3.49	3.26	2.86		2.97	2.71	2.40	4

(*p*<.001 for all differences).

**Table 4:** Preferred Societal Responses by Audience Segment.

Survey Questions	Audience Segment						Scale Points
	Alarmed	Con-cerned	Cautious	Dis-engaged	Doubtful	Dis-missive	
23. Priority of global warming for president & Congress	3.54	2.89	2.29	2.57	1.54	1.11	4
24. Corporations should do more/less to reduce warming	4.81	4.37	3.93	3.62	3.07	2.01	4
25. Citizens should do more/less to reduce warming	4.75	4.23	3.74	3.58	3.03	1.97	4
26. Desired US effort to reduce warming, given associated costs	3.78	3.33	2.89	2.83	2.01	1.37	4
27. Contingent int'l conditions for US mitigation action (% regardless of actions of other countries)	98	93	74	84	59	40	--

(*p*<.001 for all differences).

**Figure 1:**
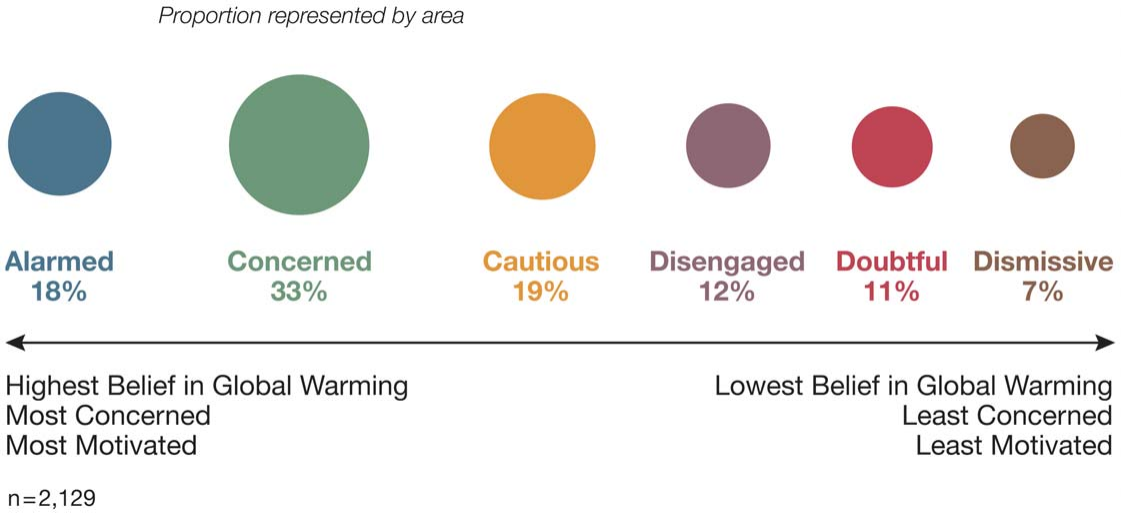
Proportion of the U.S. adult population in the Six Americas.

The six identified segments – each of which was given a concise name to summarize its essential qualities – differ dramatically with regard to what they believe about global warming, how engaged they are with the issue, what they are doing about it, and what they would like to see American government officials, businesses, and citizens do about it. The six segments also differ dramatically with regard to size: the largest represents 33% of the U.S. adult population, and the smallest only 7% (Figure 1). These six audience segments represent a spectrum of concern and action about global warming, ranging from the Alarmed (18% of the population), to the Concerned (33%), Cautious (19%), Disengaged (12%), Doubtful (11%) and Dismissive (7%).

Mean values for (or in the case of three variables, percent agreement with) each of the variables used in the segmentation analysis, by segment, are presented in Tables 1, 2, 3 and 4. The between-segment differences on all of these variables, as ascertained by ANOVA or chi-square tests, were significant at p<.001. Additional profiling information about the audience segments – i.e., how the six segments differ with regard to a range of additional relevant beliefs, behaviors (including media use), values, and demographics – is available at: http://environment.yale.edu/climate/publications/global-warmings-six-americas-2009/.

In brief, the *Alarmed* are the segment most engaged in the issue of global warming. They are very convinced it is happening, human-caused, and a serious and urgent threat. The Alarmed are already making changes in their own lives and support an aggressive national response.

The *Concerned* are also convinced that global warming is a serious problem, but while they support a vigorous national response, they are distinctly less involved in the issue, and less likely than the Alarmed to be taking personal action.

The *Cautious* also believe that global warming is a problem, although they are less certain that it is happening than the Alarmed or the Concerned. They don't view it as a personal threat, and don't feel a sense of urgency to deal with it through personal or societal actions.

The *Disengaged* haven't thought much about the issue. They are the segment most likely to say that they could easily change their minds about global warming, and they are the most likely to select the “don't know” option in response to every survey question about global warming where “don't know” was presented as an option.

The *Doubtful* are evenly split among those who think global warming is happening, those who think it isn't, and those who don't know. Many within this group believe that if global warming is happening, it is caused by natural changes in the environment, that it won't harm people for many decades into the future, if at all, and that America is already doing enough to respond to the threat.

Finally, the *Dismissive*, like the Alarmed, are actively engaged in the issue, but on the opposite end of the spectrum. The large majority of the people in this segment believe that global warming is not happening, is not a threat to either people or non-human nature, and is not a problem that warrants a personal or societal response.

To validate the predictive utility of these audience segments, we conducted four regression analyses using demographics (i.e., age, household income, gender, marital status, employment status, and race/ethnicity), political ideology, and segment membership as predictors of an outcome measure. A scale measuring support for nine specific potential federal greenhouse gas emission reduction policies was used as the outcome measure; these specific policy support measures are distinct from the preferred societal response measures used in the segmentation analysis, which are more general in nature (see Table 5). As shown in Table 6, demographics (Model 1, F = 2.8; p<.01), political ideology (Model 2, F = 267; p<.001) and segment status (Model 3, F = 1,411; p<.001) are each significant predictors of policy support when assessed in isolation of each other. Conversely, when assessed simultaneously (Model 4), demographic variables are not significant predictors, political ideology is a significant predictor with a moderately sized beta coefficient (B = .10; p<.001) and audience segment status is a significant predictor with a large beta coefficient (B = .60; p<.001). Audience segment alone explains as much variance in policy preferences (41%), as do demographics, political ideology and audience segment combined. We interpret these findings as validation of the predictive validity of the audience segmentation.

**Table 5:** Support for Emission Reduction Policies by Audience Segment.

Survey Questions	Audience Segment					
	Alarmed	Con-cerned	Cautious	Dis-engaged	Doubtful	Dis-missive
1. Establish a special fund to help make buildings more energy efficient and teach Americans how to reduce their energy use. This would add a $2.50 surcharge to the average household's monthly electric bill.	3.25	2.91	2.48	2.54	2.09	1.56
2. Provide a government subsidy to replace old water heaters, air conditioners, light bulbs, and insulation. This subsidy would cost the average household $5 a month in higher taxes. Those who took advantage of the program would save money on their utility bills.	3.44	3.07	2.81	2.79	2.23	1.78
3. Regulate carbon dioxide (the primary greenhouse gas) as a pollutant.	3.67	3.22	2.93	2.86	2.43	1.84
4. Require electric utilities to produce at least 20% of their electricity from wind, solar, or other renewable energy sources, even if it cost the average household an extra $100 a year.	3.50	3.14	2.76	2.60	2.36	2.10
5. Sign an international treaty that requires the United States to cut its emissions of carbon dioxide 90% by the year 2050.	3.51	3.07	2.64	2.68	1.98	1.49
6. Require automakers to increase the fuel efficiency of cars, trucks, and SUVS, to 45 mpg, even if it means a new vehicle will cost up to $1,000 more to buy.	3.64	3.32	3.12	2.73	2.68	2.33
7. Fund more research into renewable energy sources, such as solar and wind power.	3.84	3.57	3.31	3.16	3.14	2.96
8. Provide tax rebates for people who purchase energy-efficient vehicles or solar panels.	3.60	3.33	3.12	2.78	2.91	2.60
9. Increase taxes on gasoline by 25 cents per gallon and return the revenues to taxpayers by reducing the federal income tax.	2.50	2.14	2.00	1.97	1.69	1.37
10. Policy support index (mean of 9 measures; α = .86)	3.44	3.09	2.80	2.68	2.39	2.00

(All items measured on 4-point scales, where 1  =  strongly oppose & 4  =  strongly support; *p*<.001 for all differences).

**Table 6:** Policy Support Predicted by Socio-Demographics, Political Orientation & Audience Segment.

	Model 1:Socio-demographics	Model 2:Political orientation	Model 3:Audience segment	Model 4:Full model
Age	.01			.01
Education	.06*			.00
Household Income	.00			.01
Gender (2 = F)	.05*			-.02
Marital status (2 = married or w/partner)	-.02			.01
Work status (2 = working)	-.02			-.02
Race: white	-.14			-.07
Race: black	-.06			-.06
Race: Hispanic	-.01			-.04
Race: other	-.04			-.05
Political ideology (5 = very liberal).		.33***		.10***
Audience segment(6 = Alarmed)			.64***	.60***
Adjusted R^2^	.01	.12	.41	.41
F	2.8**	266.8***	1,411.7***	120.8***
N	2,067	2,052	2,062	2,052

*p<.05;

**p<.01;

***p<.001.

Note: Cell entries are standardized regression weights. For dummy variables, the excluded race category was “mixed race, non-Hispanic.”

To enable identification of segment status with new, independent samples, we created an identification tool based on a linear discriminant function of all 36 variables used in the segmentation analysis. This identification tool – termed the “full discriminant model tool” – correctly classified 90.6% of the sample (ranging from 79 to 99% in the six segments; see Table 7). We also developed a shorter, more practical 15-item identification tool by eliminating the 20 least predictive variables from the discriminant function. This short identification tool – termed the “reduced discriminant model tool” – when applied to our dataset, correctly classified 83.8% of the sample (ranging from 60 to 97% in the six segments).

**Table 7:** Prevalence of Audience Segments in 2008 Based on Three Methods of Identification.

Segment	Latent Class Analysis	FullDiscriminant Model		ReducedDiscriminant Model	
		Proportion of SampleIn Segment	Accuracy of Discriminant Analysis	Proportion of SampleIn Segment	Accuracy of Discriminant Analysis
1. Alarmed	18.0%	18.0%	92.6%	17.1%	85.6%
2. Concerned	33.3%	33.4%	91.3%	33.5%	85.8%
3. Cautious	18.7%	17.6%	87.5%	18.0%	80.9%
4. Disengaged	12.2%	13.6%	98.9%	14.9%	96.7%
5. Doubtful	10.6%	9.5%	79.2%	8.0%	60.1%
6. Dismissive	7.2%	8.0%	93.2%	8.5%	89.9%

## Discussion

With this research, we set out to identify and validate an audience segmentation system that can be used to inform global warming public engagement campaigns, and to develop easy-to-use survey-based identification tools that can be used to replicate our results with acceptable levels of accuracy. Both aims were achieved with a large representative sample.

To be useful in supporting public engagement campaigns, a market segmentation scheme must demonstrate five attributes: (1) segments must be distinct from one another, and members of each segment must be sufficiently similar to be effectively targeted by the same marketing strategy; (2) segments must have direct relevance to the campaign objectives being pursued; (3) segments must be large enough to justify the time and effort required to target them; (4) the segment status of individuals in the market must be identifiable; (5) the campaign organization – or organizations – must be capable of targeting one or more of the identified segments (which may involve making the necessary changes to its structure, information and decision-making systems) [19].

The audience segments we identified possess the first four of these five attributes. The six segments – all of which are substantial in size, and whose members can be identified with the tools we developed – are distinct from one another in ways that have direct bearing on efforts to promote global warming mitigation and adaptation. The last of these five attributes, ultimately, is demonstrated by whether or not campaign organizations find value in making campaign decisions using the segmentation system. In the following paragraphs, we briefly elaborate on how global warming campaign organizations might select among the six audiences identified.

Members of the *Alarmed* segment are a highly engaged and active audience, at least in their capacity as consumers (with the exception of their travel behavior, which is more-or-less similar to that of other segments). They have a strong demonstrated tendency to use their consumer purchasing power to reward businesses they believe are contributing to solutions, and punish businesses they believe are not. They are markedly less active in their role as citizens, however; only about one in four had contacted an elected official in the past year to urge them to take action to reduce global warming. Organizations seeking to promote policy advocacy – and possibly those seeking to modify people's travel behavior -- should consider targeting this audience.

Members of the *Concerned* segment are moderately engaged in the issue, but they are less active than are the *Alarmed*. As a result of their high prevalence in the population (1 out of every 3 adults), and their high stated intention to use their consumer purchasing power more frequently in the future to reward businesses they believe are contributing to solutions, organizations seeking to promote change through markets – rather than, or in addition to, change through public policy – should consider targeting this audience.

Members of the *Cautious* segment are only modestly engaged in the issue, and they don't appear ready to take action either as consumers or citizens. Organizations that are interested in expanding the number of Americans who are actively considering the issue of climate change (rather than attempting to change people's behavior, or develop support for policy responses) should consider targeting members of this audience. Narrative-based communication [20], and reframing the issue in terms of human health may be productive approaches [21].

Members of the *Disengaged* segment currently have no involvement in the issue. The *Disengaged* stand apart from other segments in that they are less educated and have lower household incomes, both of which place them at higher than average risk of being harmed by global warming [22]. This is a difficult segment to reach using news media and other traditional science communication channels, both due to their current lack of interest and their financial challenges. Organizations seeking to engage members of the *Disengaged* must think creatively about how to make the issue more relevant for them. As with the *Cautious* segment, narrative-based communication, and reframing the issue in terms of human health may be productive approaches. Activating new voices to explain the relevance of climate change – such a health professionals [21], members of the faith community [23], and organizations serving low-income families – may be helpful as well.

Members of the *Doubtful* segment are important because – although they currently doubt that global warming is real or harmful, and are disinclined to support actions to address it – they remain open to learning more about this issue. Because the *Doubtful* tend to be politically conservative, organizations that have the ability to work effectively across the political spectrum should consider developing activities to further engage the *Doubtful*.

As a result of their strongly held belief that global warming is not happening or is not human caused, members of the *Dismissive* segment are highly involved in the issue as adamant opponents to taking any form of action against global warming. Like members of the *Alarmed* segment, however, they are supportive of taking both personal and societal actions to reduce energy use. Thus, while they are likely not a productive audience for a global warming public engagement campaigns per se, they may be an attractive audience for energy-efficiency campaigns because they are receptive to such appeals.

It is important to note that the three classes of variables included in our segmentation – motivations, behaviors, and policy preferences – did not include structural and contextual factors (e.g., the availability of public transportation options, and local or state government incentives to reduce energy use) that previous research has shown to be important in influencing adoption of energy efficiency and conservation actions [24]. The implications of this decision are evident in the fact that the between segment differences on energy use and conservation actions are relatively small (albeit significant), whereas the between segment differences on global warming advocacy actions are more pronounced (see Table 3). Thus, this segmentation system is optimized for efforts to educate or engage the public about global warming per se, and less optimized for campaigns intended to promote changes in energy use behavior.

An integral part of strategic planning for a public engagement campaign involves selecting the target audiences that are the best fit for the organization's public engagement goals and resources [25]. Depending on their goals and resources, some organizations might be well served to focus their entire effort on a single target audience. Other organizations might be best served by targeting several audiences, if feasible. Regardless, campaigns that target specific audiences and tailor their materials accordingly are more likely to achieve their public engagement objectives than campaigns that do not [26].

For any given organization, the optimal target audiences are those that are likely to maximize the return on investment in campaign planning and execution. The three most relevant considerations in making that determination are the size of the audience segment, the likelihood that members of the segment will respond in the intended manner, and the organization's ability reach that segment with its current resources [27].

It remains to be seen whether or not organizations involved in global warming public engagement campaigns will be capable of – or interested in – targeting one or more of the identified segments in the ways we describe. A number science-based organizations – including science academies [28], science museums [29], and natural resource and conservation organizations – are currently considering their targeting and tailoring options using the audience segments identified. These developments may be evidence that the method possesses the final necessary attribute of utility: organizations must be capable of targeting one or more of the identified segments [19].

The “one size fits all” approach to global warming communication appears to be the default mode for most organizations, despite the fact that non-targeted approaches are at odds with best practices in campaign management [25]. National global warming education campaigns, for example, tend not to target well-defined audiences, but focus instead on the general public [9].

That non-targeted approaches remain common suggests that many organizations can't – or aren't willing to – bear the added costs of a targeted approach. Non-targeted campaigns are, without question, easier to implement than targeted campaigns. Segmenting and targeting multiple audiences can involve making changes to the organization's structure, information and decision-making systems [19]. At very least, a sustained effort to understand and engage more than one distinct target audience requires a campaign team to divide its planning and program development activities among each audience under consideration. A more intensive approach involves creating a campaign team to focus on each targeted audience [27]. These more intensive methods are common in consumer marketing organizations, yet they remain largely unknown or underutilized outside of the for-profit sector.

To monitor the stability of the audience segments identified in this research over time, we used the 36-item tool on three subsequent national surveys conducted in 2009 and 2010. Pronounced shifts in the size of the segments were evident across the three years of these surveys; for example, the Alarmed segment contracted sharply and the Dismissive segment grew markedly between fall 2008 and late 2009, but both regressed somewhat toward their prior sizes by mid-2010 [30]. We are currently exploring the reasons for these shifts, but our preliminary investigations suggest that meaningful exogenous factors – including a pronounced downturn in the economy, negative media coverage associated with the illegal release of email between climate scientists which became known as “Climategate,” and escalation of industry funded global warming denial campaigns [31] – were responsible for the shifts rather than inherent instability in the segmentation method. Indeed, two of these national surveys were conducted within one month of each other [30], [32]. Results from these surveys show only small differences between segment sizes when they are measured more-or-less contemporaneously: Alarmed, 13 vs. 14%; Concerned, 28 vs. 31%; Cautious, 24 vs. 23%; Disengaged, 10 vs. 10%; Doubtful, 12 vs. 12%; and Dismissive, 12 vs. 11%.

Our 15- and 36-item survey-based audience segment identification tools – as well as SAS & SPSS syntax to process the data – are available at: http://www.climatechangecommunication.org/SixAmericasManual.cfm. We encourage global warming campaign organizations and social science researchers to examine and evaluate them for their potential utility. To assess the robustness of this method to cultural and other contexts, we particularly encourage social science researchers to adapt these tools and assess their validity in nations other than the U.S.

## Materials and Methods

### Survey Method

In September through October of 2008, we conducted a nationally representative survey of American adults using KnowledgePanel, an online panel operated by Knowledge Networks. Recruited nationally using random-digit dialing (RDD) telephone methodology, KnowledgePanel is representative of the U.S. population. The panel tracks closely the December 2007 Current Population Survey (published jointly by the U.S. Census Bureau and the Bureau of Labor Statistics) on age, race, Hispanic ethnicity, geographic region, employment status, and other demographic variables.

The length of our questionnaire – a 50-minute completion time for the average respondent – exceeded what most respondents are willing to answer in a single session. As a result, we divided the content of the instrument into two separate questionnaires. An invitation to participate in the first survey was emailed to 3,997 randomly selected panel members. A total of 2,496 completed the questionnaires, a 62% cooperation rate. Two weeks after administration of the first survey was ended, respondents to the first survey received an invitation to participate in the second survey. Completed questionnaires were received from 2,164 respondents, an 87% cooperation rate, leading to an overall 54% within panel completion rate for the study. The period of administration for each survey – from invitation to termination of data collection – was approximately 10 days, during which one reminder email was sent to non-respondents.

To reduce the effects of any non-response and non-coverage bias in the overall panel membership, a post-stratification adjustment was applied using demographic distributions from the most recent data from the Current Population Survey (CPS). Benchmark distributions for Internet Access among the U.S. population of adults are obtained from KnowledgePanel recruitment data since this measurement is not collected as part of the CPS. The post-stratification variables were: Gender (Male/Female); Age (18–29, 30–44, 45–59, and 60+); Race/Hispanic ethnicity (White/Non-Hispanic, Black/Non-Hispanic, Other/Non-Hispanic, 2+ Races/Non-Hispanic, Hispanic); Education (Less than High School, High School, Some College, Bachelor and beyond); Census Region (Northeast, Midwest, South, West); Metropolitan Area (Yes, No); Internet Access (Yes, No).

### Measures

We measured a total of 306 variables with the two instruments; 36 of those variables were used in the audience segmentation analysis. Specifically, the 36 items were developed to assess four categories of global warming- and energy-related constructs: global warming beliefs (Table 1), global warming issue involvement (Table 2), global warming and energy efficiency and conservation behaviors (Table 3), and preferred societal response to global warming (Table 4). An index of support for nine specific federal greenhouse gas reduction policies was constructed and used to assess the validity of the segmentation results (Table 5).

### Segmentation Analysis

To identify the audience segments, the 36 variables were subjected to Latent Class Analysis using LatentGold 4.5 software [33], [34]. LCA is a modeling technique for analyzing case level data with the objective of identifying groups of respondents (segments or latent classes) with similar characteristics. LCA assigns cases into clusters using model-based posterior membership probabilities estimated by maximum likelihood methods. One advantage of LCA is it can handle nominal, ordinal, and continuous variables as well as any combination of these [33]. In addition, unlike cluster analysis, LCA is not highly sensitive to missing data. Respondents with 80% or more complete data on the 36 variables were included in the analysis; this resulted in a sample size of 2,129 for modeling purposes.

The 36 variables in our model were a mixture of ordinal and nominal variables. We submitted five, six, and seven segment solutions to the analyses. One potential problem in estimating latent class models is the possibility of obtaining a local maximum solution rather than a globally-based solution: an estimation algorithm may converge on a local maximum solution, which is the best solution in a neighborhood of the parameter space, but not necessarily the best global maximum. As models become more complex this problem increases. To guard against local maximum solutions, the estimation algorithm should be run several times with different parameter start values. Thus, to address this issue and to ensure the validity and stability of the findings, we conducted the analyses using 5,000 random sets of start values and replicated each solution to ensure model stability. All three models (5-, 6-, and 7-segments) replicated exactly. The three models had similar fit statistics (see Table 8). We examined the profile data for all three models and determined that the six-segment solution offered the highest face validity. Although the seven-segment solution had slightly lower fit statistics (which indicates a better model fit), the difference was small and the six segments described above were more interpretable.

**Table 8:** Model Fit Statistics.

	L^2^	BIC(L^2^)	Npar	P(L^2^)
5 classes	146560.858	133330.136	402	<.0001
6 classes	145443.384	132695.443	465	<.0001
7 classes	144595.960	132330.799	528	<.0001

To create an easy-to-use tool for others to categorize survey respondents in new, independent samples, we created a linear discriminant function using the output from the Latent Class Analysis. In contrast to Latent Class Analysis, discriminant analysis does not permit missing data. We therefore used mean substitution for missing data, and then applied this linear function using all 36 variables to our data set. The 36 variable linear function correctly classified 90.6% of the sample (ranging from 79 to 99% in the six segments; see Table 7). Elsewhere in this paper we refer to the 36-variable linear function as the “full discriminant model tool.”

### Brief Screening Tool Development

To develop a shorter – and therefore more easily used – screening questionnaire capable of classifying members of independent samples into the six audience segments with 80% accuracy or better, we eliminated the 21 least predictive variables from the discriminant function. The resultant 15-item “brief” screening instrument, when applied to our dataset, correctly classifies 83.8% of the sample (ranging from 60 to 97% in the six segments; see Table 7). Elsewhere in this paper we refer to the 15-variable linear function as the “reduced discriminant model tool.”

### Validation of the Segments

To validate that the segments account for variance in important measures above and beyond variance accounted for by other common explanatory measures, we conducted a series of linear regressions. The dependent measure for these analyses was an index of support for a series of nine federal policies that, if enacted, should reduce national levels of greenhouse gas emissions. Responses to each of these questions were combined into a simple index; the Cronbach's alpha for this policy support scale was 0.86.

In the first analysis, the demographic variables of age, gender, income, education, marital status, work status, and race were regressed against the GHG reduction policy support measure. In the second analysis, a five-point political ideology scale (very liberal, somewhat liberal, moderate, somewhat conservative, very conservation) was added into the regression. In the final analysis, audience segment status was added into the regression.

### Human Subjects Approval and Informed Consent

This research was approved by the Human Subjects Review Board at George Mason University and Yale University. Written informed consent was obtained from all participants involved in this research.
